# Late Danubian mitochondrial genomes shed light into the Neolithisation of Central Europe in the 5^th^ millennium BC

**DOI:** 10.1186/s12862-017-0924-0

**Published:** 2017-03-16

**Authors:** Maciej Chyleński, Anna Juras, Edvard Ehler, Helena Malmström, Janusz Piontek, Mattias Jakobsson, Arkadiusz Marciniak, Miroslawa Dabert

**Affiliations:** 1 Department of Human Evolutionary Biology, Institute of Anthropology, Faculty of Biology, Adam Mickiewicz University in Poznań, Umultowska 89, 61-614 Poznań, Poland; 2 Institute of Archaeology, Faculty of History, Adam Mickiewicz University in Poznań, Umultowska 89D, 61-614 Poznań, Poland; 3 Department of Organismal Biology and SciLifeLab, Uppsala University, Norbyvägen 18C, SE-752 36 Uppsala, Sweden; 4 Molecular Biology Techniques Laboratory, Faculty of Biology, Adam Mickiewicz University in Poznań, Umultowska 89, 61-614 Poznań, Poland; 5 Department of Biology and Environmental Studies, Faculty of Education, Charles University in Prague, Magdalény Rettigové 4, 116 39 Prague, Czech Republic

**Keywords:** Ancient DNA, Mitochondrial DNA, U5 haplogroup, Danubian Neolithic, Neolithic transition

## Abstract

**Background:**

Recent aDNA studies are progressively focusing on various Neolithic and Hunter - Gatherer (HG) populations, providing arguments in favor of major migrations accompanying European Neolithisation. The major focus was so far on the Linear Pottery Culture (LBK), which introduced the Neolithic way of life in Central Europe in the second half of 6th millennium BC. It is widely agreed that people of this culture were genetically different from local HGs and no genetic exchange is seen between the two groups. From the other hand some degree of resurgence of HGs genetic component is seen in late Neolithic groups belonging to the complex of the Funnel Beaker Cultures (TRB). Less attention is brought to various middle Neolithic cultures belonging to Late Danubian sequence which chronologically fall in between those two abovementioned groups. We suspected that genetic influx from HG to farming communities might have happened in Late Danubian cultures since archaeologists see extensive contacts between those two communities.

**Results:**

Here we address this issue by presenting 5 complete mitochondrial genomes of various late Danubian individuals from modern-day Poland and combining it with available published data. Our data show that Late Danubian cultures are maternally closely related to Funnel Beaker groups instead of culturally similar LBK.

**Conclusions:**

We assume that it is an effect of the presence of individuals belonging to U5 haplogroup both in Late Danubians and the TRB. The U5 haplogroup is thought to be a typical for HGs of Europe and therefore we argue that it is an additional evidence of genetic exchange between farming and HG groups taking place at least as far back as in middle Neolithic, in the Late Danubian communities.

**Electronic supplementary material:**

The online version of this article (doi:10.1186/s12862-017-0924-0) contains supplementary material, which is available to authorized users.

## Background

The Danubian Neolithic is a sequence of archaeological cultures that emerged around 5600/5500 BC in the Transdanubia region of western Hungary from the preceding Vinca, Starcevo and Köros cultures [1]. The Neolithisation of Central Europe by people of the Linear Pottery Culture, better known as the Linearbandkeramik culture (LBK), is a well-investigated phenomenon, both archaeologically and genetically. Nowadays it is agreed by both archaeologists [2, 3] and geneticists [4–7] that the rapid spread of the LBK in the second half of 6th millennium BC was one of the major migration events that shaped the European gene pool.

The archaeologists see the LBK as one of the stages of migration process originating in Near East, that spread north- and west-wards gradually introducing the Neolithic way of life in Europe [8]. However some archaeological data also points to involvement of local Hunter-Gatherers (HGs) in the formation of the LBK and its eastern variant the Alföld Linear Pottery (ALP) culture from Starčcevo and Körös cultures [9]. The relatively rapid pace of the spread of the LBK in Central Europe in the second half of 6th millennium BC, together with different, in comparison with local HG groups, economy and material culture, is being interpreted as an evidence of migratory character of this movement [10]. Based on archaeological data it has been suggested that LBK populations either replaced the pre-existing HG populations or, alternatively, coexisted with them utilizing different resources and areas [11, 12]. However some degree of contact between sites representing both the LBK and HG context is observed in form of exchange of goods such as lithic resources [13]. The LBK presence in Central Europe lasted for around four hundred years, and throughout this period it remained seemingly unchanged maintaining its economic, cultural and social character [14]. In the first half of 5th millennium BC this homogenous culture differentiated into several distinctive cultures belonging to late Danubian sequence (LDN in our paper). Those new cultural entities include: Grossgartach, Rössen, Stroke-ornamented ware culture (StBK), Late Band Pottery Culture (LBPC), Malice, and Lengyel cultures, and are found in more diverse set of ecological zones compared to the LBK [15]. They also display a changing socioeconomic organization of their communities [14]. In some regions, notably in the northern part of former LBK occupation, large stable settlements were replaced by small and short-lived sites with late Danubian archaeological assemblages, located on sandy-soils occupied earlier by local HG. The post-LBK period—the first 400–500 years after the end of the LBK—in all the main regions of the Polish lowlands marked a complete disintegration of the preceding LBK arrangements and the discontinuous development of new forms of spatial organization. The changes are thought by archaeologists, to originate from internal social dynamics of those communities, although they allowed for more extensive contacts with HG groups in later period [15]. The second half of 5th millennium BC marks the emergence of the final groups belonging to the Danubian Neolithic, with the Brześć Kujawski Group being the prominent example in the Polish lowlands. Archaeologists see these final stages of Linear Pottery cultures as a synthesis of various elements, including those of local HG both in terms of economy and material culture [16]. The end of Danubian sequence is marked by another widespread phenomenon known as the Funnel Beaker Culture (TRB, from Trichterbecherkultur). This new entity emerged almost simultaneously along Baltic Coast and in the Kuyavia region in the end of the 5th millennium BC. The TRB is sometimes called Northern Europe’s first autochthonous farming population [17]; however the idea is being disputed as early TRB exhibits very few Mesolithic elements in their archaeological record. Moreover, since it was partially contemporaneous with the LDN sequence, the emergence of the TRB as a result of divergence of linear cultures, strengthened by different interregional contacts, cannot be excluded [13].

Geneticists have been trying to directly address questions related to the origins and spread of Neolithic populations in Central Europe. The investigation of hypervariable region (HVR) of mitochondrial (mt) DNA from LBK individuals from present-day Germany, Austria and Hungary [18, 19], led researchers to propose the N1a, later supplemented by T2, J, K, V and HV [19, 20] as a ‘signature haplogroups’ of the LBK, as they were found in relatively high frequencies in early farmers in comparison to HG and modern European populations. By contrast haplogroups U2, U4, U5 and U8 have been found to dominate in European HG populations [21–25] differentiating them from LBK communities. Further data from LBK populations, (including ALP) and their predecessors Starčevo and Körös cultures, show similar haplogroup patterns to that reported for the LBK alone, pointing to genetic uniformity of linear cultures and lack of HG genetic involvement in their formation [5, 26].

The genomic data supported the idea about lack of genetic continuity between the LBK and the local Hunter - Gathers [5, 27, 28]. However, the LDN populations such as the Rössen culture have received less attention and are often merged and/or analyzed together with data from individuals from LBK contexts [29]. The contacts between Danubian and HG communities that led to gradual change of their economy and material culture seen by archaeologists seem to have no impact on the genetic composition of both communities. However, sparse occurrences of typical HG haplogroups found in late Danubian populations [30, 31], indicate that some degree of genetic influx occurred between those populations. The samples coming from the TRB complex also were the focus of the researches, who found out that they genetically differed slightly from LBK individuals [20, 22], with whole genome data showing higher HG component in the TRB [6].

The main aim of our study was to analyze genetic affinities of LDN populations looking for potential genomic influx from HGs. We generated complete mt genomes from individuals belonging to LBK and late Danubian contexts excavated in modern-day Poland and combined these data with available partial and complete ancient European mt genomes to investigate haplogroup differences between LBK, its successors from the LDN sequence, Central European Hunter-Gatherers and other post-Danubian populations.

## Methods

### Sample selection

Twenty-five individuals were selected for our study of mt genomes. The individuals had been excavated in either a LBK context (*n* = 7) or various LDN contexts, including Lengyel culture (*n* = 12), Brześć Kujawski Group (BKG; *n* = 4) and Malice Culture (MC; *n* = 1). Additionally, one Mesolithic individual from Janisławice in central Poland was included. Detailed archaeological information for all the individuals can be found in supplementary material (Additional file 1: Supplementary Text S1 and Additional file 2: Table S1).

For comparative population analyses, we used either published mt haplogroup frequencies (*n* = 992) or, where available, complete mitochondrial genomes retrieved from web depositories http://www.ncbi.nlm.nih.gov/genbank, http://www.ebi.ac.uk/ena; (*n* = 192). For analyses based on haplogroup frequencies, we grouped available data following [32] with minor modifications (Additional file 2: Table S2). We merged all LDN mt haplogroup data and excluded HG data predating last glacial maximum following results by [33] to better represent the local genetic makeup at the time of arrival of the first farmers. We also added Near Eastern Neolithic groups based on recently published data [27, 34].

**Fig. 1:**
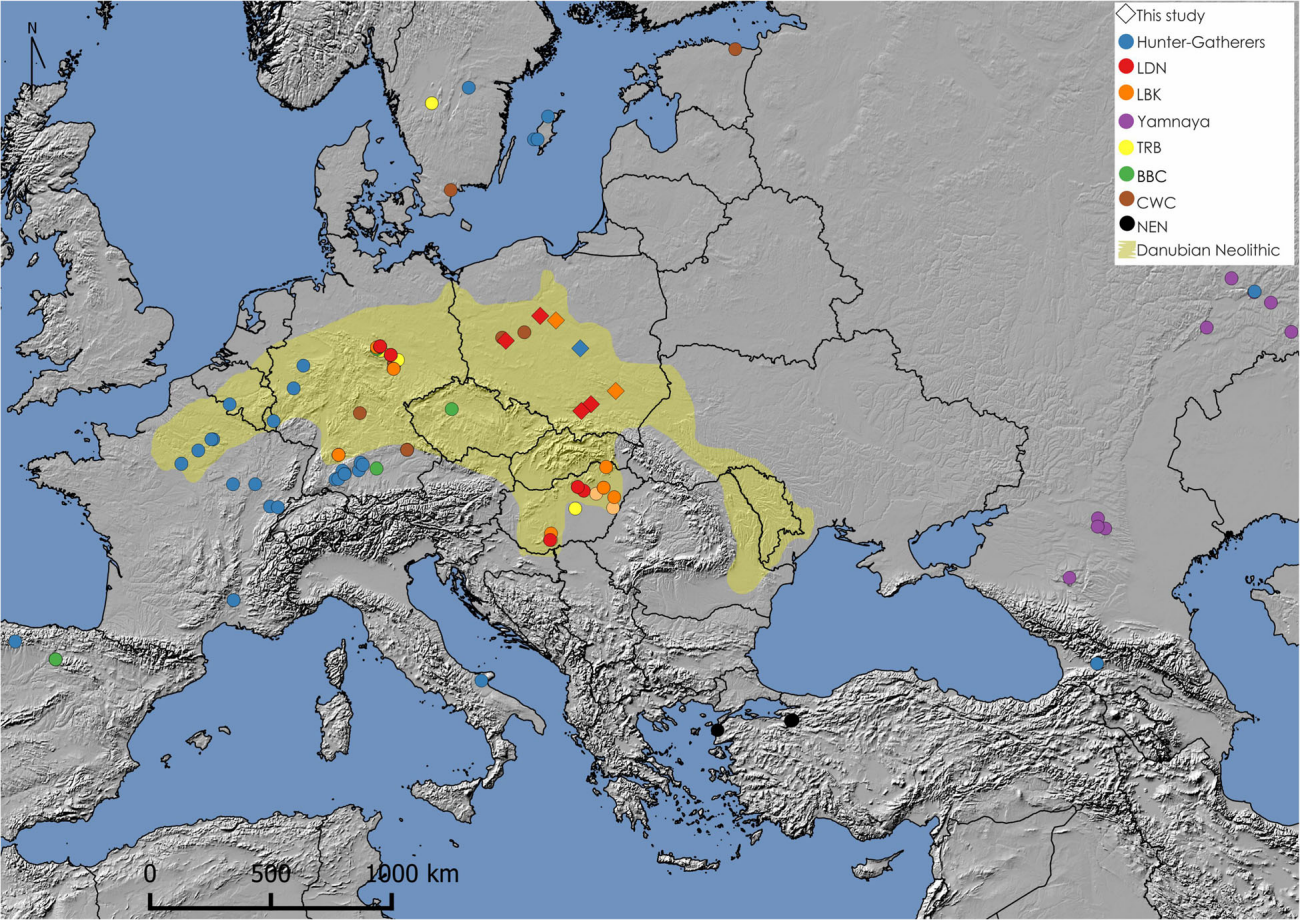
Location of samples with complete mitochondrial genomes used in the study. Genomes produced in this study (*diamond shape*); genomes retrieved from previously published data (*circles*); maximum extent of Danubian Neolithic cultures highlighted in *yellow*; NEN - Near East Neolithic; LBK - Linear Pottery Culture; BBC - Bell Beaker Culture; TRB - Funnel Beaker Culture; CWC - Corded Ware Culture; YAM - Bronze Age Yamnaya; HG - Hunter - Gatherer; LDN - Late Danubian cultures

The complete mitochondrial genomes were divided into eight distinct groups, including Hunter-Gatherers (HG), Near Eastern Neolithic (NEN), Yamnaya and major central European Neolithic cultures where all data for populations associated with the Funnel Beaker Culture were merged into one group (TRB) (Additional file 2: Table S3). The location of individuals with complete mitochondrial genome data used for this study, including our newly sequenced samples, is shown in map generated with QGIS 2.12.2 (Fig. 1).

### DNA extraction

All necessary precautions against DNA contamination were used during the collection of samples and extraction of aDNA. From each individual either teeth or fragments of cortical bone from long bones were collected and analyzed.

Extraction of aDNA was conducted in the laboratory designed for aDNA analysis at the Faculty of Biology, Adam Mickiewicz University in Poznań. The laboratory is equipped with positive air pressure, HEPA filters, flow hoods and automatic UVC lamp system. Before DNA extraction, teeth and bone fragments were cleaned with 5% NaOCl and rinsed three times with sterile water followed by UV irradiation for at least two hours per each side. After UV exposure, the samples were drilled using Dremel® drill bits and ~250 mg of teeth/bone powder was collected to sterile screw cap tubes (2 ml). The inner part of teeth roots and cortical bone of long bones were targeted. Total DNA was extracted from the powder using a silica-based method [35] modified as in [36].

### DNA library preparation and sequencing

Twenty microliters of DNA extract were converted into blunt-end Illumina genomic library, following [37], omitting the initial nebulization step due to fragmentation of a DNA. Genomic libraries were amplified by setting up 6 individual PCR reactions for each library as in [38]. All six PCR reactions per library were pooled and purified with AMPure® XP Reagents (Agencourt-Beckman Coulter) following manufacturer’s protocol. The concentrations of the libraries and DNA fragment length distributions were calculated using High Sensitivity D1000 Screen Tape assay on 2200 TapeStation system (Agilent Technologies). Prior to sequencing, up to12 indexed DNA libraries were pooled in equimolar amounts and sequenced together. The libraries were sequenced on an Illumina HiSeq 2500 run in 125 pair-end mode at the SNP & SEQ technology platform in Uppsala, Sweden.

### mtDNA capture

The DNA libraries that yielded insufficient mitochondrial genome coverage (<9 X) through the initial Illumina screening and generated more than 25 mitochondrial reads were enriched for mtDNA sequences, amplified and sequenced as in [31]. This was done by using an in-solution hybridization capture method and commercially biotinylated DNA-capture MYbaits® probes (MYcroarray®) following the manufacturer’s instructions. Two rounds of capture were performed for each sample [39]. Fusion primers PISI and AIS4 [31] were used for the second round of post-capture amplification, enabling sequencing of blunt-end Illumina libraries on Ion Torrent PGM system (Life Technologies). The sequencing was performed at the Molecular Biology Techniques Laboratory, Faculty of Biology, Adam Mickiewicz University in Poznań, according to manufacturer’s instructions.

### Sequence analyses

To process Illumina’s HiSeq2500 shotgun sequencing data, we followed the procedure described in [38]. MergeReadsFastQ_cc.py [37] script was used to remove adapters and merge read pairs, requiring an overlap of at least 11 bp and summing up base qualities. Merged reads were mapped as single-end reads against the revised Cambridge Reference Sequence (rCRS) using BWA aln version 0.7.8 [40] with the non-default parameters -l 16500 -n 0.01 -o 2 -t 2. The molecular sex of the analyzed individuals was assigned based on the ratio of reads (with mapping quality greater than 30) mapping to Y and X chromosomes (R_y_) [41].

Ion Torrent PGM data from the mtDNA capture was processed as follows. The scripts fastx_barcode_splitter.pl and fastx_trimmer (from the FASTX toolkit) were used to demultiplex the reads by barcode, using a one mismatch threshold. Cutadapt v1.8.1 software [42] was then used to trim adapters using the following parameters − e 0.3333, −m 35, −M 110 − q 20, −n 5. The filtered reads were checked with FastQC v 0.11.3 [43] before being mapped against the rCRS using TMAP v3.4.1 [44] with the options: −g 3 -M 3 -n 7 -v stage1 –stage-keep-all map1 –seed-length 12 –seed-max-diff 4 stage2 map2 –z-best 5 map3 –max-seed-hits 10. FilterUniqueSAMCons.py [37] was used to collapse PCR duplicate reads with identical start and end coordinates, for both Illumina and PGM data.

All Illumina and PGM data for each sample were then merged using samtools merge option [45]. Misincorporation patterns and fragment length distribution were analyzed using mapDamage v2.0.5 [46]. Additionally we used Schmutzi software [47] with default settings to estimate present-day human contamination levels by scanning a database of modern human putative contamination sources.

The Biomatters IGV software v2.3.66 [48] was used to visualize the sequence assembly. Finally, consensus sequences were built using the program ANGSD v0.910 [49] and only reads with minimum base quality of 20, mapping score of 30 and a minimum coverage of 3x were used. Previously published mt genomes were reconstructed from BAM files acquired from published data using the same pipeline as described above.

Mitochondrial haplotypes were determined based on the PhyloTree phylogenetic tree [50] build 17 and by using the HAPLOFIND web application [51]. The mutations reported as missing or unexpected were visually inspected in IGV to check if they could be results of misincorporations in low coverage regions. In one case, the sample from Janisławice (Jan1), the coverage was insufficient for reliable consensus sequence assignment. Here, the BAM file was inspected visually in order to detect SNPs and only SNPs that were supported by reads with a total coverage of 3x and containing reads from both forward and reverse strands were reported.

### Population genetic analyses

We have computed PCA using RapidMiner Studio 7 (RapidMiner Inc., Boston, MA, USA). The PCA results and variable haplogroup loadings were plotted using Matplotlib 1.5.1 Python package/MS Excel/. To keep the PCA results graph transparent, we only display loadings of the 10 most important haplogroups that predominantly influence the PCA scores.

To explore the clustering properties of PCA results based on haplogroup frequencies without the bias of knowing the archaeological background of the populations, we have used the k-means clustering method on the PCA results. After testing several configurations with k = {4, 5,…, 9}, the best clustering for our dataset according to Davies Bouldin Index [52] was obtained using Squared Euclidean distance, with k = 6. The k-means clustering was performed in RapidMiner Studio 7.

Pairwise genetic distances were computed in the Arlequin 3.5 software [53] using the complete mitochondrial genomes of the ancient individuals. To reduce the chance of incorporating erroneous SNPs, we have trimmed the first and last 30 nucleotides from all of the consensus mtDNA sequences, because these regions were ambiguously sequenced in some reference data. We computed Nei’s average number of pairwise differences between the populations [54] with 1000 permutations and p-value of 0.05 and used its linearized form [55].

Standard version of analysis of molecular variance (AMOVA) [53] was used to calculate the proportion of genetic variance between and within the defined groups. In the Arlequin 3.5 package, the AMOVA was computed from the pairwise F_ST_ matrix (10 000 permutations). This test was primarily used to infer the affiliation of populations into broader defined clusters of populations. AMOVA was used to assess the likelihood that any of the other cultures was a continuous population with LDN, a total of 8 population structures were tested. LDN was grouped consecutively with one from the other 7 populations (HG, LBK, TRB, NEN, CWC, BBC, YAM) while remaining 6 populations were kept separately (Additional file 2: Table S6).

The pairwise genetic distances between populations were used to compute the multidimensional scaling (MDS). Python scikit-learn 0.17 package [56] was used to compute the MDS.

With the use of the Networks 4.614 software (fluxus-engineering.com) we have calculated the median networks. To reduce possible bias and noise in the data, we have removed all polymorphic positions with unknown SNPs (“Ns”). Following [57, 58], the network construction procedure included weighting the most common mutations inversely according to their frequency. The batches of most frequent polymorphisms were assigned reduced weights using in-house script (deposited at https://github.com/EdaEhler/bioutils_python) in the following manner: the most frequent polymorphism (or group of polymorphisms) was given the weight of 1, the second most common polymorphism was given the weight of 2, the third most common polymorphism was given the weight of 3, etc. up to 10. All remaining polymorphic loci retained the default weight of 10. Computations consisted of using Reduced Median algorithm [57] followed by Median Joining algorithm [58] and maximum parsimony calculation (postprocessing) to reduce the superfluous links [59].

## Results

### Sequencing and mitochondrial haplotypes

Out of 22 samples screened by shotgun sequencing, only one yielded enough data to reconstruct the whole mitochondrial genome and assign haplogroups (Additional file 2: Table S1). From the remaining samples, 8 more libraries that produced at least 25 mitochondrial fragments were selected for the mtDNA enrichment process. The average mitochondrial genome coverage after capture varied between 2 and 113 and therefore we could determine the haplogroups for 4 more of the samples. New mitochondrial genomes published in this study, together with partial genome from Jan1 individual are presented in Table 1. Additional information on sequencing and enrichment efficiency can be found in Additional file 2: Table S4.

**Table 1:** Five Neolithic complete mitochondrial genomes acquired in this study and one Mesolithic Hunther-Gatherer sample with determined mitochondrial haplotype

Sample	Archaeol. culture	Region	Site	Age*	Morphological sex	mt genome coverage	% of mt genome^a^	Haplotype
Jan1	HG	Kuyavia	Janisławice	5509 ± 135 cal. BC	M	23x	84,4116	U5b1b1
Sam1	LBK	Little Poland	Samborzec	*5300–5000 BC*	F	34x	99,7888	N1a1a1a
KM1	LDN	Little Poland	Kazimierza Mała	*4800–4500 BC*	F	27x	99,9759	U5b1b
KZ6	LDN	Kuyavia	Krusza Zamkowa	*4500–4000 BC*	F	43x	99,9215	N1a1a1a3
R18_1	LDN	Greater Poland	Racot	4200 ± 55 cal. BC	F	9x	98,6119	K2a
NHP1	LDN	Little Poland	Krakow Nowa Huta Pleszów	*3800–3600 BC*	-	113x	99,9940	H5

^a^percent of nucleotide positions covered by at least 3 reads of sufficient quality

*normal font - C14 dates, italic typochronological dating

The nucleotide misincorporation patterns of the mitochondrial sequences were assessed using the mapDamage software and showed fragment length distribution and the deamination patterns characteristic for aDNA: C to T transitions accumulated at 5′ ends and corresponding G to A transitions at 3′ ends of DNA fragments (Additional file 3: Figure S1). The contamination estimates acquired with Schmutzi software supported this result as in all samples they were below 4% (Additional file 2: Table S4). In total, we acquired 5 new mitochondrial genomes for Danubian Cultures from contemporary Polish lands: 1 from the LBK and 4 from LDN Lengyel and Malice Cultures and partial genome from HG individual from Janisławice. All genomes are deposited in GenBank under accession numbers KY091894-KY091898. Mutations against the RSRS as reported by Haplofind and used for haplogroup assignment are listed in Additional file 2: Table S5. The LDN individuals belonged to N1a1a1a3, K2a, H5 and U5b1b haplogroups. The LBK individual belonged to N1a1a1a. Based on partial mitochondrial genome data, the Mesolithic individual from Janisławice belonged to haplogroup U5b1b1.

**Fig. 2:**
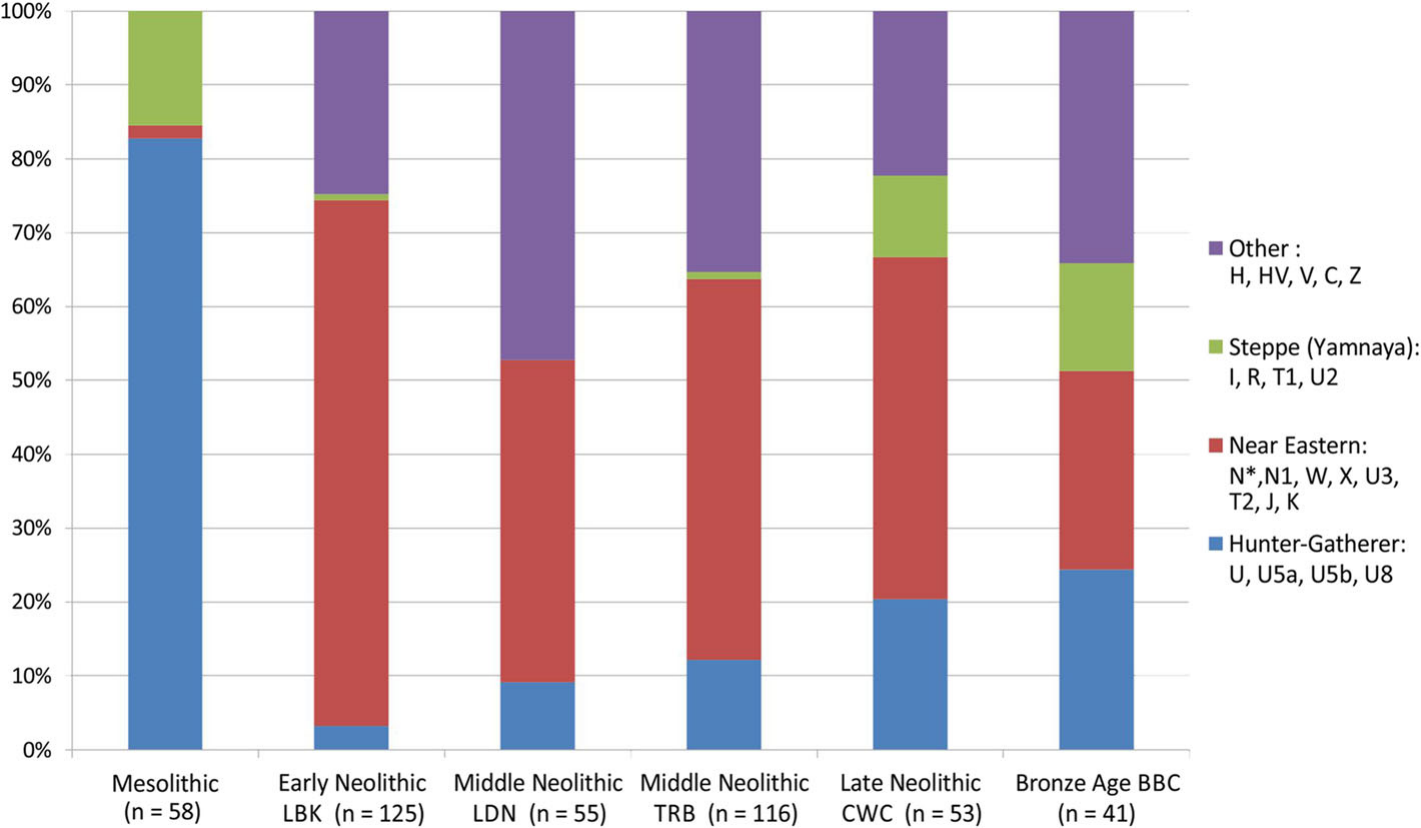
The frequencies of mitochondrial haplogroups grouped in major components of European gene pool. LBK - Linear Pottery Culture; TRB - Funnel Beaker Culture; CWC - Corded Ware Culture; BBC - Bell Beaker Culture; LDN - Late Danubian Neolithic

Figure 2 shows haplogroup frequencies where haplogroups were combined together into European gene pool components following the division made by [20]. When pooled with all available reference data, the acquired haplogroups indicate around 10% higher than previously reported frequency of HG component in late Danubian populations.

### Population genetics

**Fig. 3:**
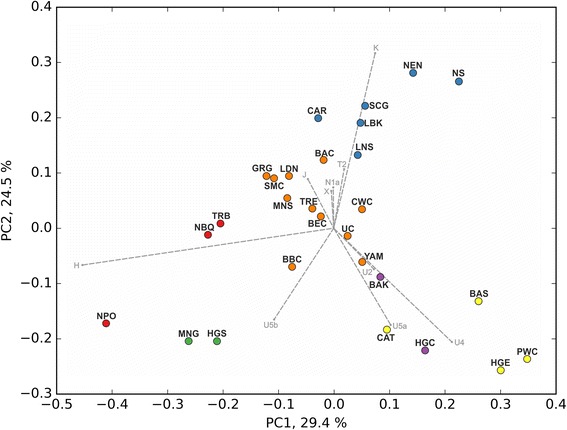
PCA plot with k-means clustering (k-value = 6), the colors depicts 6 generated clusters. HGC - Hunter - Gatherer central; LBK - Linear Pottery Culture; SCG - Schöningen group; BAC - Baalberge Culture; SMC - Salzmünde Culture; BEC - Bernburg Culture; CWC - Corded Ware Culture; BBC - Bell Beaker Culture; UC - Unetice Culture; MNG - Middle Neolithic Germany; TRB - Funnel Beaker Culture; PWC - Pitted Ware Culture; HGS - Hunter-Gatherer south; CAR - (Epi)Cardial; NPO - Neolithic Portugal; NBQ - Neolithic Basque Country & Navarre; MNS - Middle Neolithic Spain; TRE - Treilles; HGE - Hunter-Gatherer east; BAS - Bronze Age Siberia; BAK - Bronze Age Kazakhstan; CAT - Catacomb Culture; YAM - Bronze Age Yamnaya; NEN - Near East Neolithic; NS – Neolithic Syria; GRG - Gurgy ‘Les Noisats’ group; LNS - Late Neolithic/Chalcolitic Spain; LDN - Late Danubian cultures

The PCA plot of the two first principal components, describing 29.4 and 24.5% variability respectively (Fig. 3), shows that LDN samples are in close vicinity of other early and middle Neolithic samples, however further from the LBK than expected. Instead LDN is much closer to some populations belonging to the TRB complex, i.e. Salzmünde (SMC) and Baalberg (BAC) cultures. It is also in direct proximity to Middle Neolithic Spain (MNS), Gurgy (GRG) and Treilles (TRS) populations. The k-means clustering always grouped the LDN within a cluster of middle Neolithic cultures, including the MNS, GRG and BAC. Depending on k - value the composition of this cluster changed but it always contained the LDN, LBK and BAC populations. Figure 3 shows the combined results of PCA with color coding according to clustering with k value of 6. The displayed PCA loadings show that the most influential haplogroups having effect on PCA scores, are U4, U5a, U5b, U2, T2, K, and H.

**Fig. 4:**
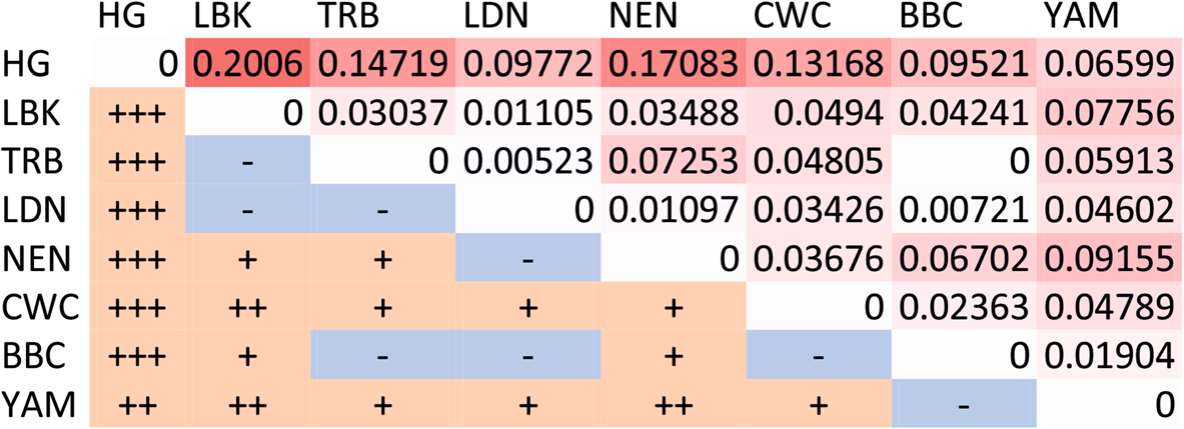
F_st_ Matrix. Above diagonal: F_st_ values; below diagonal: p-values (significance level: <0.0500 (+), <0.0050 (++), <0.0005 (+++)). NEN - Near East Neolithic; LBK - Linear Pottery Culture; BBC - Bell Beaker Culture; TRB - Funnel Beaker Culture; CWC - Corded Ware Culture; YAM - Bronze Age Yamnaya; HG - Hunter - Gatherer; LDN - Late Danubian cultures

The F_ST_ values with Slatkin [55] linearization applied, obtained from complete mitochondrial genomes are shown in Fig. 4. The results indicate that the LDN have close genetic affinity with both LBK (F_ST_ = 0.01105) and TRB (F_ST_ = 0.00523) populations as well as with BBC (0.00721) population. At the same time the Hunter-Gatherers are set apart from late Danubians (F_ST_ = 0.09772).

**Fig. 5:**
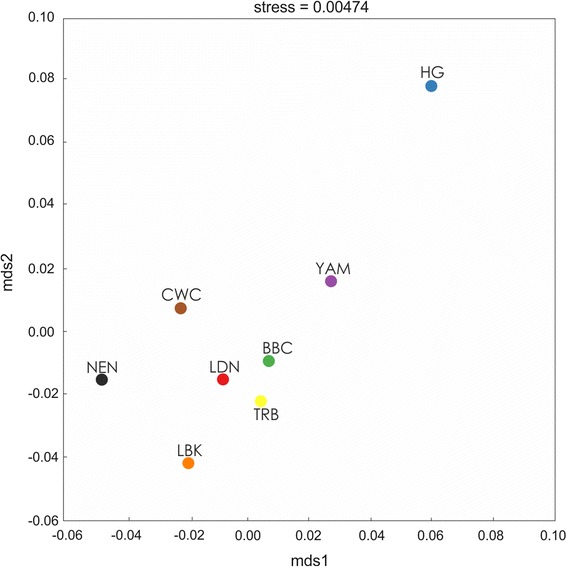
MDS plot of F_ST_ values. NEN - Near East Neolithic; LBK - Linear Pottery Culture; BBC - Bell Beaker Culture; TRB - Funnel Beaker Culture; CWC - Corded Ware Culture; YAM - Bronze Age Yamnaya; HG - Hunter - Gatherer; LDN - Late Danubian cultures

The F_ST_ based MDS plot (Fig. 5) yields similar results to the PCA, with the LDN being in closer proximity to the TRB, than to the LBK.

AMOVA results (Additional file 2: Table S6) also point to closer relation of late Danubians to the TRB (within group variation of −0.88 and among groups variations of 8.87) than the LBK (0.66 and 7.5, respectively) populations.

**Fig. 6:**
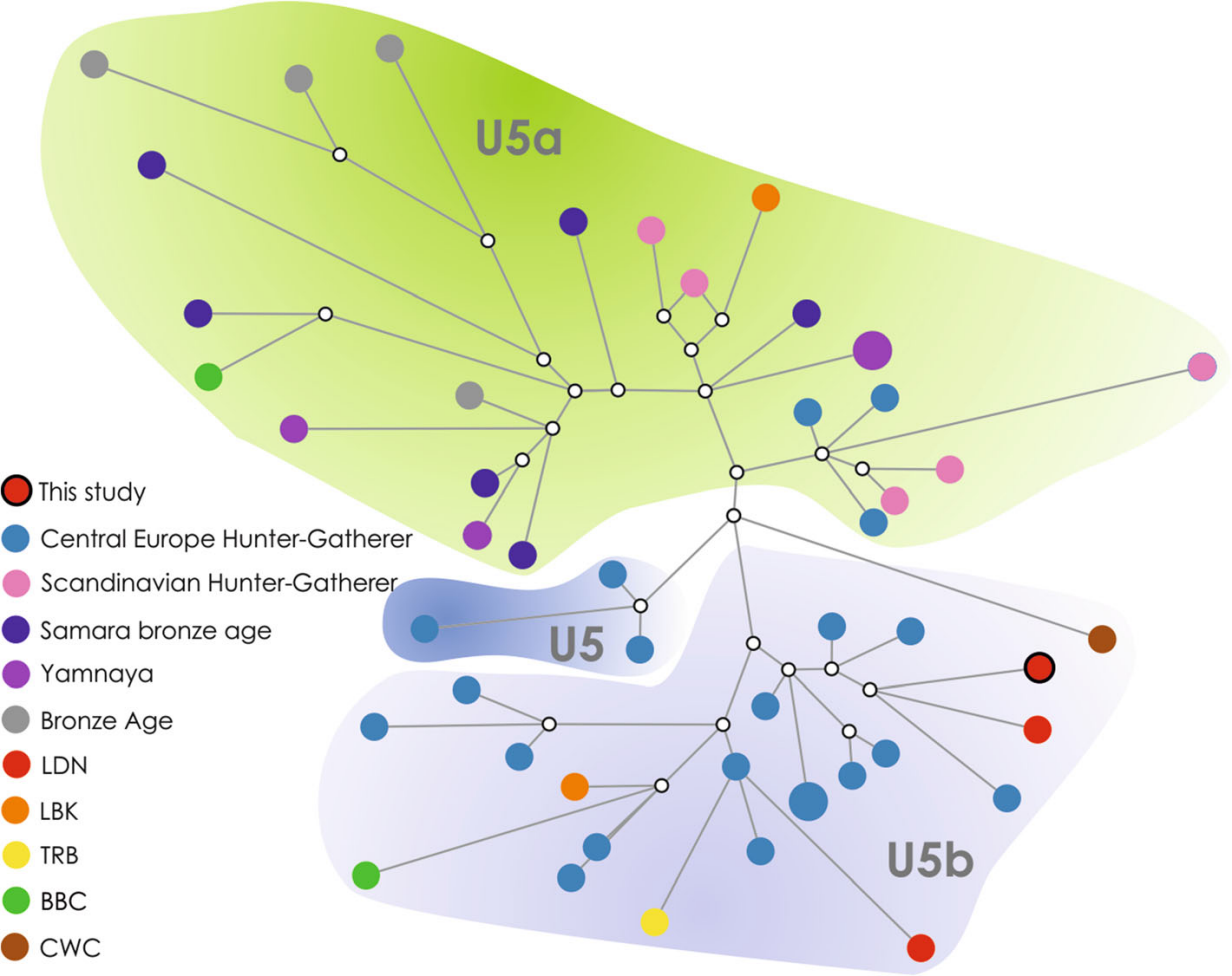
The median network for haplotypes belonging to U5 haplogroup. Linear Pottery Culture LBK; Funnel Beaker Culture TRB; Corded Ware Culture CWC; Bell Beaker Culture BBC; Bronze Age Yamnaya YAM; Late Danubian cultures LDN

The median network generated for U5 haplotypes show that haplotypes belonging to U5b found in early to late Neolithic communities from Central Europe, including LDN U5b1b haplotype discovered in this study, all originate from various Central European HG haplotypes (Fig. 6).

## Discussion

Our data indicate close relation of the LDN to various middle and late Neolithic populations, especially those belonging to the TRB complex. This stands in contradiction to suggestions that late Danubian populations should be in closest genetic relation to the LBK, the notion previously suggested by both archaeologists [2] and geneticists [20]. Our results also contradicts the hypothesis, that TRB groups belonged to a different tradition than the Danubian sequence, and that they had other, possibly local Mesolithic, origins [17, 60]. Instead our data support the idea that through their long presence in Central Europe, the Danubian populations gradually differentiated from groups associated with the LBK culture into a sequence of populations belonging to various LDN populations, and that some of those populations in turn were driving the emergence of the TRB [13].

The majority of the haplotypes from LDN populations, acquired by us and in other studies, are considered to be typical for the LBK. However, several of them belong to U5b and U5a haplogroup [30, 31, 61] which is thought to be a part of HG component of the European gene pool. The relatively similar frequency of U5b haplogroup, found in populations belonging to both the LDN and the TRB complex seems to be responsible for drawing them closer together in our analyses. The haplogroups U5b and U5a are considered to belong to Hunter-Gatherer component of European gene-pool [20]. The origin of U5b in TRB populations from modern-day Germany is being interpreted as an increase in Hunter-Gatherer mt lineages in middle Neolithic [20], a view supported by whole genome data [32]. Likewise, based on whole genomic data, some genetic admixture from Scandinavian HG to northern TRB populations was observed [6].

Neither PCA nor MDS can directly answer where U5 individuals, differentiating LDN and TRB from LBK, came from, and what was the direction and nature of the gene flow. The Hunter - Gatherers of Central Europe, based on well documented history of contacts between them and LDN communities, seem to be most probable candidate for source of U5 haplotypes. On the other hand, HG populations are also frequently documented to live close to farming communities but maintaining their genetic distance [23]. One can also argue that TRB populations lived in close proximity with late Danubian communities (in particular the Brześć Kujawski Group [62] and therefore the possibility of a gene flow from the TRB to the LDN should also be taken into consideration.

However, the network analysis shows that both LDN and TRB U5b haplogroups originated from haplotypes found in HG individuals, so the direct gene flow between LDN and TRB groups, based on few available samples, is not supported. Notably, in case of U5b part of the median network all basal (i.e. close to the center of the network) haplotypes belong to Central Europe HG while the haplotypes found in LDN, TRB, LBK, BBC and CWC samples form the outer part of the network, which suggests that they constitute a derived version of haplotypes, with more accumulated substitutions compared to the U5b haplogroups from HG.

Assuming that HG groups were the source of U5 haplotypes in LDN populations it would be interesting to speculate on exact source and nature of this gene flow. The fact that out of four U5 individuals found in LDN populations 3 belong to U5b while one belongs to U5a haplogroup [61]. One possible explanation of this disproportion is the low sample size and the U5a might simply have been missed by our sample. However, what is noteworthy the majority of Mesolithic individuals with U5a haplotypes come from outside of Central Europe, most notably from Scandinavia [6, 22]. That stands in contrast with the fact that a majority of cultural exchange, between the LDN and HG, is seen between the LDN and northern HG groups (notably Ertebølle culture) and not neighboring lowlands groups (such as Janisławice culture) [15]. Meantime, HG elements seen in the TRB material culture came from various HG backgrounds, both from Baltic see coast, and European lowlands [13]. However, the cultural diversity of European Hunter - Gatherers does not seem to be reflected by their genetic diversity [33], as they seem to be rather uniform group and therefore differentiating between potential sources of genetic influx to either the LDN or the TRB might be difficult.

The two new U5b haplotypes, recovered in this and our previous study [31], come from two geographically and chronologically distant sites. U5b2a1a was found in KZ1 female from the Brześć Kujawski group of Lengyel Culture. It is dated to 4226 ± 74 cal. BC, and comes from Krusza Zamkowa site, which is known for its rich burials with numerous imports [63], some stylistically resembling HG artifacts. U5b1b found in the KM1 female excavated in Kazimierza Mała site belonged to the Malice Culture dated to around 4800–4500 BC. The Malice Culture itself exhibits influences from other linear pottery groups mostly from the Lengyel and Polgár cultures south from it [64]. Elements of Hunter-Gatherer origin are not proposed for the Malice Culture itself; however close ties and its possible involvement in formation of the Brześć Kujawski group are debated [65].

Our data, from this and the previous study [31], shows that in its long existence the LBK gradually changed into the LDN not only in terms of material culture but also in terms of its genetic composition. The change observed by us in form of introduction of U5b haplotypes is most probably the result of gene flow from HG populations. This idea, proposed also for populations from western fringe of LDN populations [30], pushes back in time the resurgence of HG maternal lineages seen by others in TRB populations [20]. The nature of this flow might be, to some extent, explained by what we know about social and migration behaviors of Danubians. Both isotopic and genetic data have given us some clues about the social organization of early farming communities. Strontium isotope data suggest greater mobility of women than men in some LBK populations [66, 67], which may indicate patrilocal model of society. The genetic data also points towards patrilocality in Early Neolithic as a most probable cause of differences in mtDNA and Y-chromosome composition of European gene pool [68]. This hypothesis is also strengthened by much higher haplogroup diversity of mtDNA than Y-chromosome DNA observed in the LBK population from Hungary [26]. Similar higher diversity of maternal compared to paternal lineages was found in Neolithic populations that emerged along Mediterranean route of neolithisation [69, 70]. Those results may explain the nature of influx of foreign mitochondrial haplogroups into early farming communities. It seems possible that to some extent it was customary to introduce females from neighboring communities into LBK and LDN groups, with different Hunter-Gatherer groups being the most probable source of this influx. However, to determine that more, both genetic and isotopic data is needed from those populations.

## Conclusions

We acquired 5 new complete mitochondrial genomes from Late Danubian populations from modern day Poland. Population genetic analyses run on our data combined with published haplotypes show that Late Danubian populations are maternally closer related to TRB groups instead of culturally similar LBK. We assume that U5 haplotypes found by us and other researchers in those populations are responsible for this affinity. Since U5 is thought to be one of haplogroups characteristic for HG component of European gene pool, we argue that its presence points to gene flow from HG to both Late Danubian and TRB populations.

## Additional files

Additional file 1: Supplementary Text S1. Archaeological information on samples used in the study. (PDF 888 kb)
Additional file 2: Table S1–S6. S1 List of all ancient individuals analyzed in the study and results of library screening, S2 Ancient comparative populations used in PCA and their mitochondrial haplogroups, S3 List of individuals used for F_ST_ and MDS, S4 Sequencing results of analyzed samples used for reconstruction of mitochondrial genomes, S5 Mutations against the RSRS as reported by haplofind found in acquired mitochondrial genomes, S6 results of AMOVA. (XLS 185 kb)
Additional file 3: Figure S1. Deamination patterns: frequency of C to T transitions (red) at the 5′ends of reads (left) and frequency of G to A transitions (blue) at the 3′ends of reads (middle). and fragment length distribution of sequenced libraries (right). (JPG 974 kb)

## Abbreviations

aDNA: Ancient DNA
MDS: Multidimensional scaling
mtDNA: Mitochondrial DNA
PCA: Principal component analysis
PCR: Polymerase chain reaction
rCRS: Revised Cambridge reference sequence
RSRS: Reconstructed Sapiens Reference Sequence
SNP: Single nucleotide polymorphism
