# Supplementary material for: Late Danubian mitochondrial genomes shed light into the Neolithisation of Central Europe in the 5th millennium BC
Source: BMC Evol Biol. 2017 Mar 16;17:80. doi: 10.1186/s12862-017-0924-0 (PMC5356262; doi:10.1186/s12862-017-0924-0)
Supplement: Additional file 1: Supplementary Text S1. — Archaeological information on samples used in the study. (PDF 888 kb) [file 12862_2017_924_MOESM1_ESM.pdf]

Late Danubian mitochondrial genomes shed light into the Neolithisation of Central Europe in the 5th millennium BC

Maciej Chyleński, Anna Juras, Edvard Ehler, Helena Malmström, Janusz Piontek, Mattias Jakobsson, Arkadiusz Marciniak, Miroslawa Dabert

Additional file 1 Supplementary Text S1

Samples used in the study

1. Mesolithic samples (*n* = 1)
   1.1. Janisławice (sample id Jan1)

The Mesolithic grave at Janisławice, was discovered by accident in 1936 and was the only feature found on the site (with the exception of other burial frond nearby, but interpreted as coming from early modern times).
The burial had rested inside a pit of an elongated oval shape. His head was to the East-North-East, right leg flexed lightly, heels turned in. Most of the grave goods had become displaced as the upper part of the burial pit was truncated together with the upper part of the skeleton. It is thought that the upper part of the skeleton was in a vertical position as the dead individual had been buried in a seated position, the grave offerings were found next to his left arm.
The grave held 42 flint finds as well as 54 bone and antler objects, 43 of them worked, and a few fragments of mollusc shell. Some of the finds presumably belonged to a necklace [1].
The individual is interpreted as male, with an estimated height of 165 cm. The individual was 30-40 years old as determined from suture epiphyseal and cranial fusion.
The remains are dated to 5644-5374 cal BC (95.4%) [2].
Two fragments of long bones were sampled for the study.

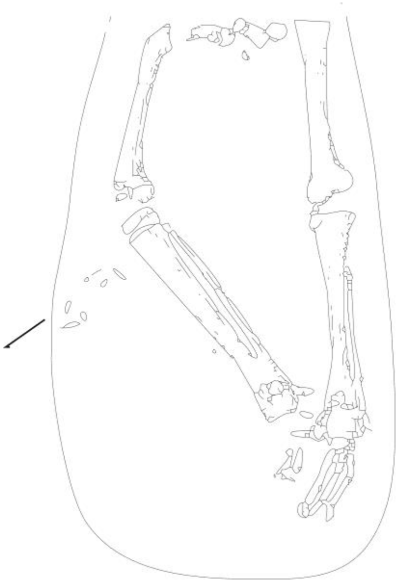

Figure 1. The outline of the undisturbed lower part of man from Janisławice [1].

2. Linear Pottery Culture (LBK) samples (*n* = 7)

2.1. Samborzec (*n* = 3)

Samborzec is a large site with many phases of occupation excavated between 1960 and 1966. One of those phases was a typical LBK settlement composed of long houses and three burials scattered around the settlement [3].

Grave 1 / Feature no. 60 (sample id Sam1)
Flexed individual placed on N-S axis and facing E deposited in rectangular pit.
The burial goods contained small round amphora with so called musical note pattern placed in the individual hands, one complete and one fragmented bone tool.
The relatively well preserved remains are interpreted as belonging to women ca. 50 years old and 155.9-159.5 cm high [3].
Two teeth: lower left P1 and upper left I2 were sampled for the study.

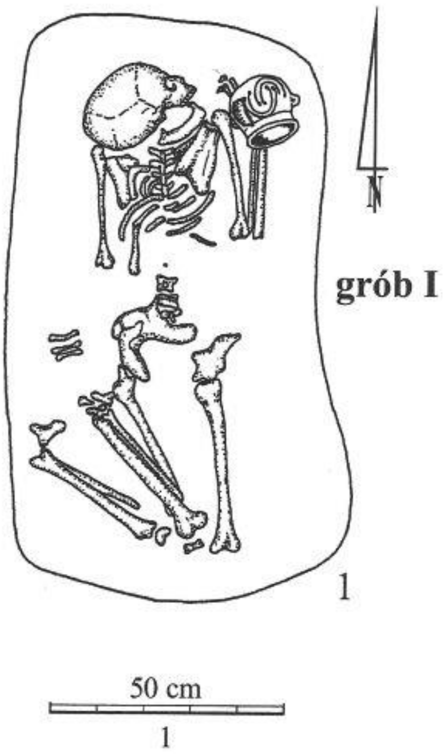

Figure 2. The burial 1 (Feature no 60) from Samborzec [3].

Grave 2 / Feature no 71 (sample id Sam2)
The irregular burial pit was badly truncated by later intrusions and therefore skeleton was disarticulated an incomplete.
Small ceramic cup with 3 lugs was found among the remains.
The incomplete skeleton belonged to an infant ca. 2 years old [3].
The fragment from the shaft of humerus was selected for the study

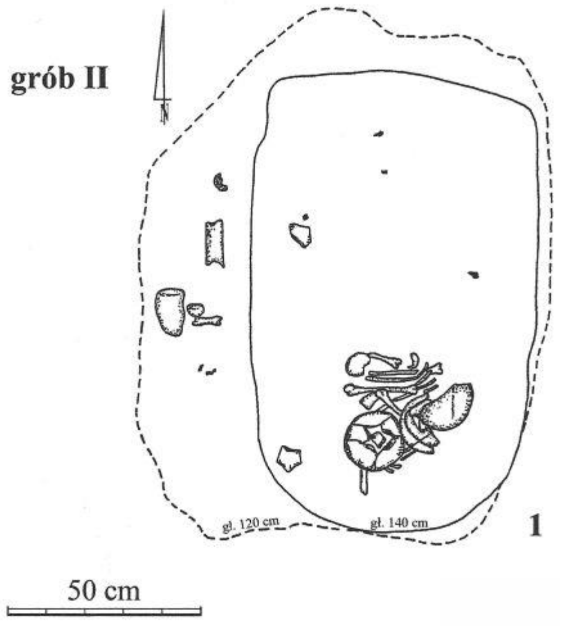

Figure 3. The burial 2 (Feature no 71) from Samborzec [3].

Grave 3 / Feature no 208 (sample id Sam3)
Flexed individual placed on its left side on W-E axis and facing S deposited in rectangular pit.
The individual was decorated with ochre and multiple mussel and limestone beads. Additional fragments of LBK pottery were also found in the burial fill.
The relatively well preserved remains are interpreted as belonging to ca.156.3-159.5 cm high adult women [3].
Two fragments of metatarsals were sampled for the study.

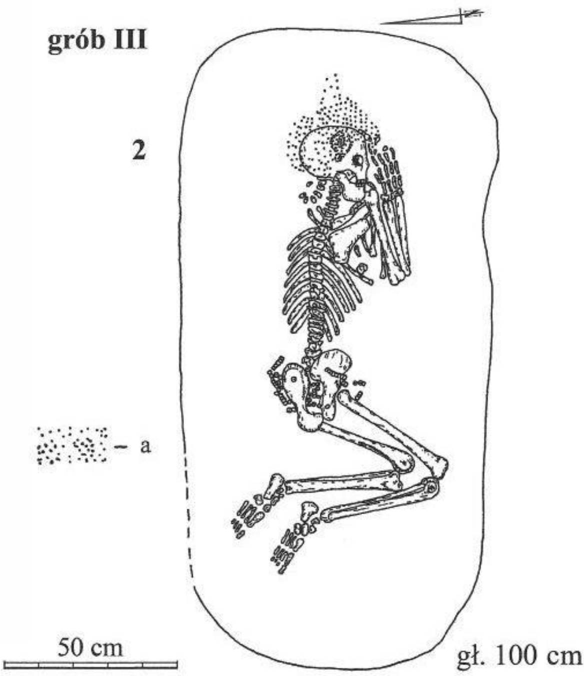

Figure 4. The burial 3 (Feature no 208) from Samborzec [3].

2.2. Ludwinowo 6 (n = 1)

Ludwinowo 6 (sample id L6_1) is a multilevel site with at least 5 phases of occupation excavated between 2008 and 2009. One of those phases was medium sized LBK settlement composed of long houses. Moreover two burials interpreted as belonging to LBK were found on site. One of which (Feature C46) was included in our study.
Badly truncated by Iron Age intrusion oval burial located among other LBK features.
The remains were poorly preserved and are interpreted as belonging to adult women around 20-30 years old. Two teeth were selected for the study.

2.3. Kruszyn 13(*n* = 2)

Kruszyn 13 is a multilevel site with at least 9 phases of occupation excavated between 2004 and 2009. One of those phases was medium sized LBK settlement composed of at least 3 households. Two burials were found within one of those household complexes [4].

Feature G107 (sample id K13_1)
Flexed individual placed on left side on W-E axis and facing E was deposited in rectangular pit.
Fragments of LBK pottery, some of which ornamented with musical note pattern, were found in the burial fill.
The relatively poorly preserved remains are interpreted as belonging to male ca. 40-50 years old.
The remains were dated to 4662 ± 51 BC, however the date comes from carbonate fraction of bone and therefore is not reliable [4].
Two teeth were selected for the study

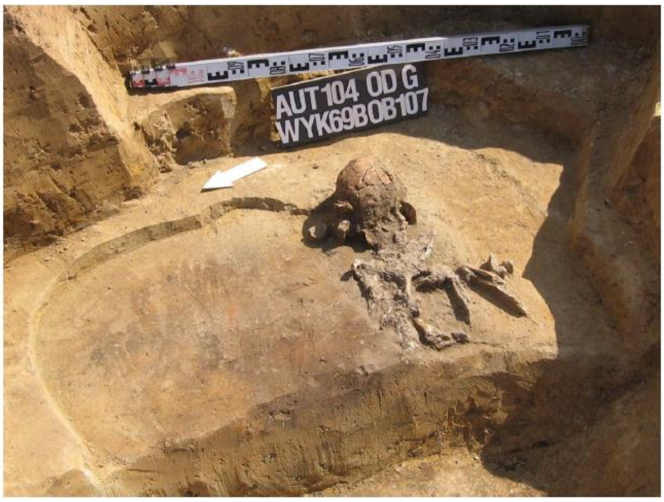

Figure 5. The burial G107 from Kruszyn 13 site [4].

Feature G110 (sample id K13_2)
Flexed individual placed on left side on NW-SE axis with head in NW was deposited in rectangular pit.
Fragments of LBK pottery were found in the burial fill.
The relatively poorly preserved remains are interpreted as belonging to 5-7 years old child.

The remains were dated to 4758 ± 38 BC, however the date comes from carbonate fraction of bone and therefore is not reliable [4].
A petrous fragment of temporal bone was selected for the study

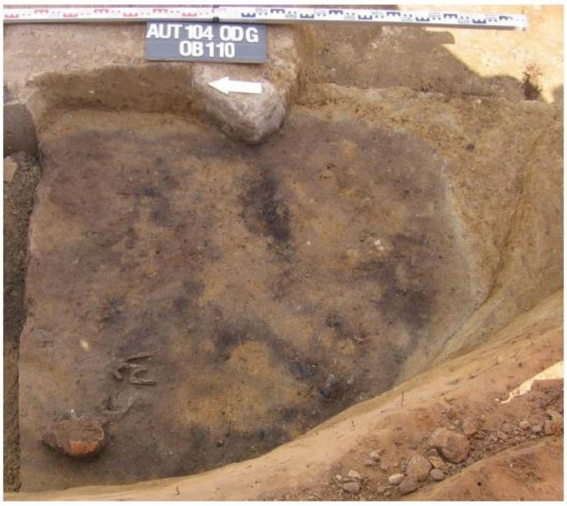

Figure 6. The burial G110 from Kruszyn 13 site [4].

2.4. Szczotkowice (*n* = 1, sample id Scz1)

The burial was discovered by accident in 1961 and was completely destroyed upon its discovery.
The burial shape and position are only known through verbal account of its finders it was supposedly placed on W-E axis with head on W.
The burial goods contained: 2 small ceramic vessels, one with musical note pattern ornament; 3 shell and 36 marble beads; unknown clay object and an animal bone.
The only surviving bone was fragment of cranium covered with ochre belonging (based on suture fusion) to an adult at least 50 years old [3].
The available fragment of cranium was sampled for the study.

3. LDN Lengyel Culture samples (*n* = 14)

3.1. Kraków Nowa Huta Pleszów (*n* = 6)

The site is located on loess terrace occupied for majority of prehistory and was excavated betwwen 1953 and 1981. One of 5 levels of its occupation is being interpreted as large Lengyel settlement with multiple burials belonging to that culture [5], 6 of which were included in our study.

Feature 818 (sample id NHP1)
Burial placed in a storage pit. The individual was seemingly lying on its right side on E-W axis with its head in W.
The burial contained two small ceramic vessels and one copper pedant. Numerous flint objects and pottery fragments were also found in a fill of the storage pit.
The remains belonged to child 1-2 years old, however a fragment of cranium belonging to adult male was also found among the human bones retrieved from the feature [5].
One deciduous incisor was sampled for our study.

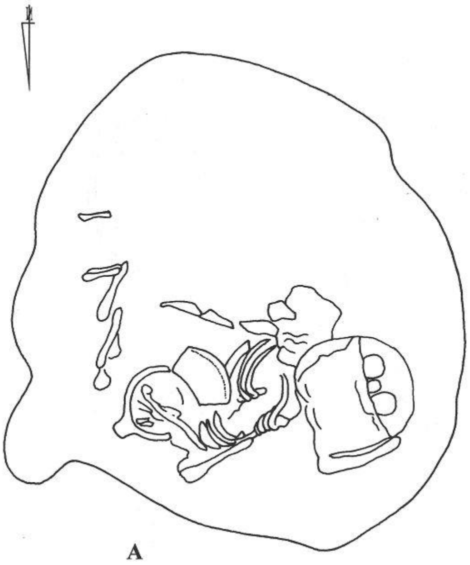

Figure 7. The burial Feature no. 818 from Kraków Nowa Huta Pleszów [5].

Feature 825 (sample id NHP2)
Flexed individual placed on right side and its head in S and facing E deposited in oval pit.
The burial goods contained 4 small ceramic vessels, 2 of which were ornamented typically for Lengyel Culture. Additionally one flint scraper and stone axe were also deposited in the burial.
The relatively poorly preserved remains are interpreted as belonging to ca. 30-50 years old individual, of hard to define sex [5].
Two fragments of long bone shafts were sampled for our study.

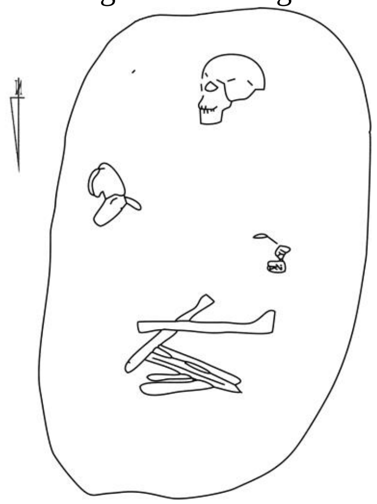

Figure 8. The burial Feature no. 825 from Kraków Nowa Huta Pleszów [5].

Feature 917 (sample id NHP3)
Flexed individual placed on left side lying in N-S axis and facing W.
Small amphora with traces of red and white paint was found within the burial.
The relatively poorly preserved remains are interpreted as belonging to ca. 35-55 years old, possibly male individual [5].
Two teeth were sampled for our study.

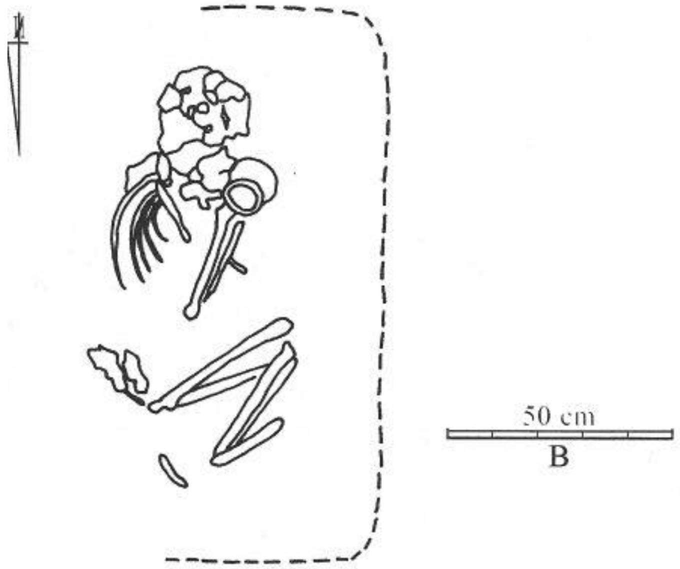

Figure 9. The burial Feature no. 917 from Kraków Nowa Huta Pleszów [5].

Feature 950 (sample id NHP5)
The burial was badly truncated by later activities. Based on the scattered remains, the individual was lying in flexed position on its left side.
The burial also contained ceramic vessel ornamented with lugs in its upper part.
The poorly preserved remains are interpreted as belonging to ca. 25-40 years old women [5].
Two fragments of long bone shafts were sampled for our study

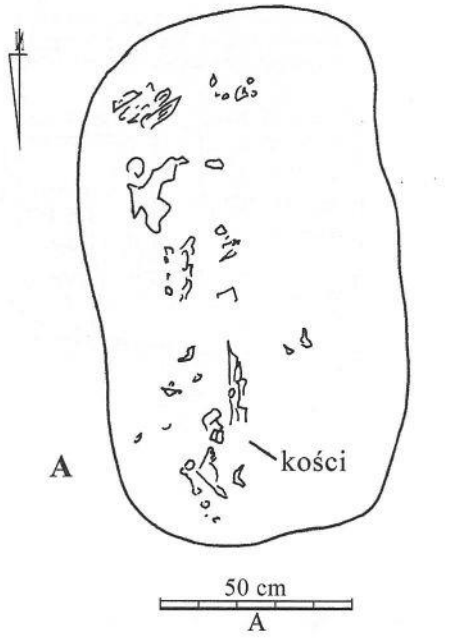

Figure 10. The burial Feature no. 950 from Kraków Nowa Huta Pleszów [5].

Feature 995 (sample id NHP6)
The burial was one of two placed in elongated pit. It was located in the S part of the pit end and was lying on its left side with head in S facing W.

Two small ceramic vessels were found in close vicinity of the individual.
The remains belong to 25-35 years old male individual [5].
Two fragments of long bone shafts were sampled for our study

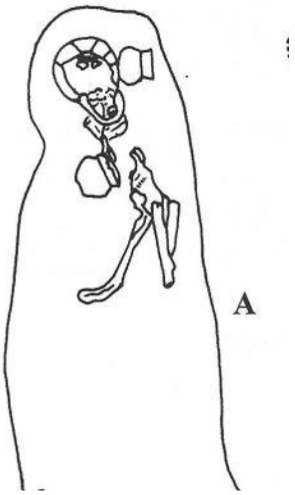

Figure 11. The burial Feature no. 995 from Kraków Nowa Huta Pleszów [5].

Feature 1338 (sample id NHP7)
Flexed individual placed on right side and its head in S and facing E deposited in oval pit.
The burial goods contained 8 flint flakes and a stone axe.
The remains belong to 20-30 years old male individual [5].
Two canine teeth were sampled for the study.

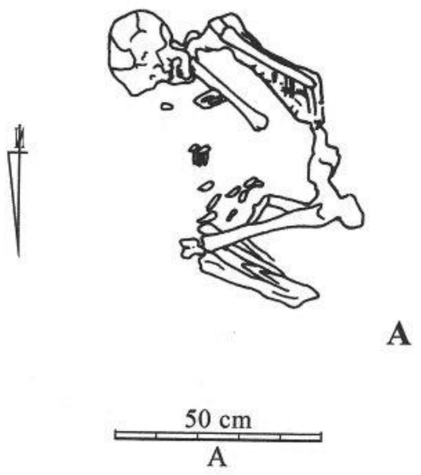

Figure 12. The burial Feature no. 1338 from Kraków Nowa Huta Pleszów [5].

3.2. Kraków Nowa Huta Cło (*n* = 2)

Kraków Nowa Huta Cło is a site with many phases of occupation lasting from Neolithic to early Middle Ages. The site was first excavated in 1950, however the burials included in this study ware all excavated in current ongoing project that started in 1997. One of phases is being interpreted as Lengyel settlement with 3 burials being interpreted as belonging to that culture [5], 2 of which were included in this study.

Feature 48 (sample id NHC1)
Burial placed in a storage pit. The individual was lying on its right side on SW-NE axis with its head in SW.
Multiple finds (pottery fragments and flint tools) associated with Lengyel culture were found in the fill of the overlying storage it.
The remains belonged to 20-30 years old probably male individual [5].
Two teeth were sampled for our study

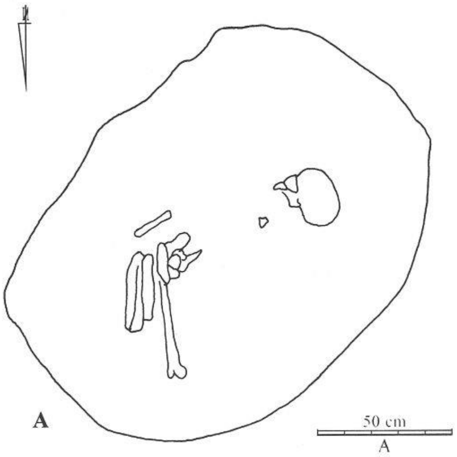

Figure 13. The burial Feature no. 48 from Kraków Nowa Huta Cło [5].

Feature 232 (sample id NHP2)
Poorly preserved remains were put in a hard to define pit. The individual were probably placed in tightly flexed position on its right side with its head in SSE.
The remains were accompanied by flint blade and small ceramic vessel with pierced lugs.
The state of the remains makes it hard to determine the sex and age of the individual [5].
Two fragments of long bone shafts were sampled for our study

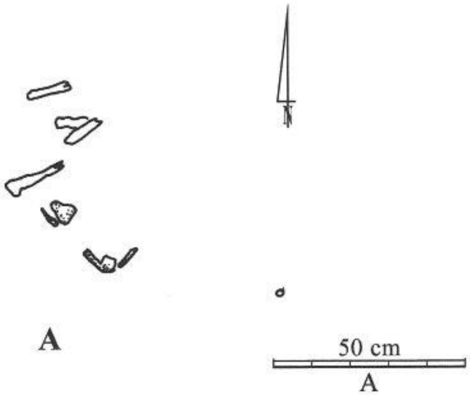

Figure 14. The burial Feature no. 232 from Kraków Nowa Huta Cło [5].

3.3. Kraków Nowa Huta Wyciąż (*n* = 4)

The site is thought to be Lengyel culture extramural cemetery. Part of which was excavated between 1950 and 1952, unearthing 13 Lengyel burials [5], 4 of which were included in our study.

Feature 25 (sample id NHW1)
Flexed individual lying in E-W axis and facing N.
The remains were accompanied by 2 ceramic vessels, one of which was a hollow pedestal bowl, and two copper objects, a bracelet and a piece of copper wire.
The remains are interpreted as belonging to ca. 30-40 years old woman [5].
Two teeth were sampled for our study.

Feature 33 (sample id NHW2)
Flexed individual placed lying in NE-SW axis and facing N.
The burial goods contained 2 ceramic vessels, one flint tool, one spindle whorl/clay bead and 3 copper objects including dagger, ornament resembling an epaulet and a bracelet made from copper wire [5].
Fragment of long bone shaft was sampled for our study.

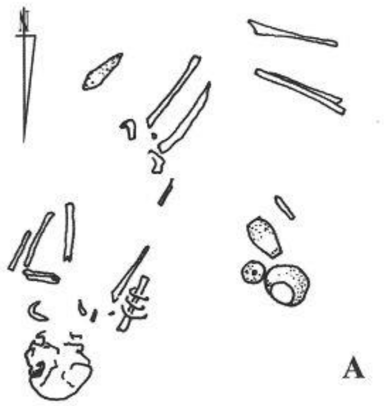

Figure 25. The outline of burial 33 from Kraków Nowa Huta Wyciąż [5].

Feature 258 (sample id NHW3)
The individual was lying on its right side on SW-NE axis facing S in a flexed position.
Between its head and knees 4 ceramic vessels were placed, one of the vessels was a hollow pedestal bowl.
The remains belonged to 25-35 years old male [5].
Two teeth were sampled for our study.

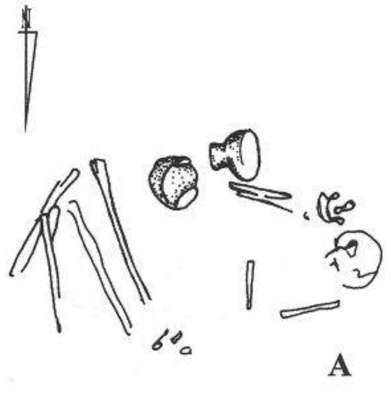

Figure 16. The burial Feature no. 258 from Kraków Nowa Huta Wyciąż [5].

Feature 259 (sample id NHW4)

The individual was lying in a flexed position, on its right side, on SW-NE axis and was facing S.

South from the remains the burial goods were placed. The assemblage contained 4 ceramic vessels, flint tool two pedants from animal canines and a clay bead.

The remains belonged to 25-35 years old male [5].

Two teeth were sampled for our study.

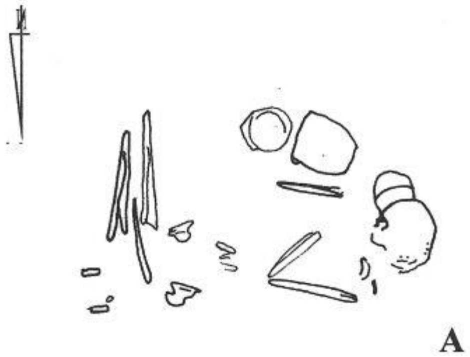

Figure 17. The burial Feature no. 259 from Kraków Nowa Huta Wyciąż [5].

4. LDN Brześć Kujawski Group/Culture (*n* = 4)

4.1. Krusza Zamkowa 3 (*n* = 1, KZ6)

Krusza Zamkowa is a site with multiple levels of occupation on of which one was large BKK settlement with several richly decorated burials. The first excavations at this site took place in 1973–1974 and 1976–1977 the current excavation project started in 2013. Five of the burials were published previously by us as a part of independent kinship study. In here we supplemented the data with another individual from Feature no 380 unearthed during first excavation [6].

The individual was placed on right side in flexed position in N-S axis with its head to S in a rectangular pit truncated by later intrusion

The skeleton was accompanied by 11 shell pedants.

The remains belonged to 25-30 year old woman [7].

Two teeth were sampled for our study.

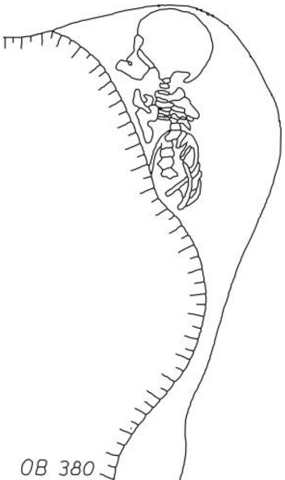

Figure 18. The burial Feature no. 380 from Krusza Zamkowa 3 site [6].

4.2. Racot 18 (*n* = 1, R18_1)

The site is being interpreted as a settlement attributed to Brześć Kujawski Group (BKG) of Lengyel Culture (sometimes treated independently as Brześć Kujawski Culture). The settlement consisted of at least 15 households representing several phases of the site occupation and was excavated in 1984-7. Among the features associated with households one richly decorated burial (Feature 82) was found [8].

The individual was lying in flexed position on its right side on N-S axis with its head to the S and facing E

The burial goods consisted of two ceramic vessels, a necklace of animal teeth and copper beads, 18 animal bone epaulets and hip belt from shells.

The remains belonged to 30-35 year old woman.

The absolute date of the burial is 4255–4145 cal BC [9].

One tooth and one petrous fragment of temporal bone were included in the study.

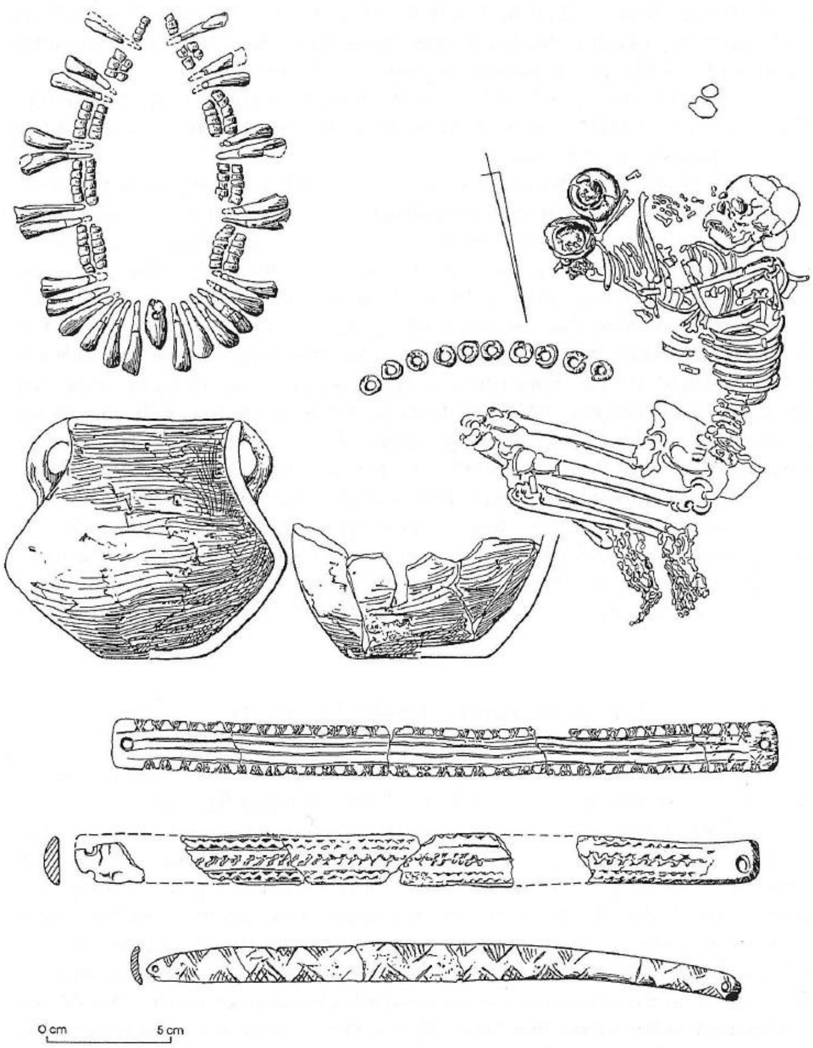

Figure 19. The outline of burial Feature no. 82 and finds associated with it, from Racot 3 site [8].

4.3. Ludwinowo 7 (*n* = 2)

Ludwinowo 7 is a multicultural site with long history of occupation, the two earliest phases are interpreted as large LBK settlements replaced later by smaller BKG settlement. The site was excavated during four seasons 2000, 2001, 2008 and 2009. There were two poorly preserved burials attributed to the BKG found on site.

Feature C3 (sample id L7_1)

Tightly flexed individual placed on left side on N-S axis and head to the S was deposited in round pit, badly damaged by post deposition activities. However it seems that some kind of structure in a form of burial chamber might have been built around the remains.

Two fragmented ceramic vessels with stroked ornament were also found within the burial.

The relatively poorly preserved remains are interpreted as belonging to female ca. 45-55 years old.

The absolute date of the feature is 4460BC (95.4%) 4330 cal BC [10].

Petrous part of temporal bone was sampled for our study.

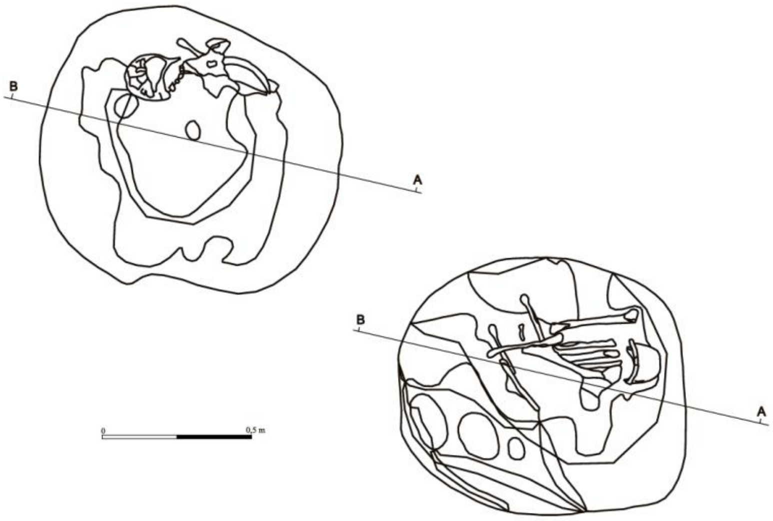

Figure 20. The burial Feature no. C3 from Ludwinowo 7 site [10].

Feature E14 (sample id L7_2)

Despite being found within LBK feature the absolute dating points towards the burial belonging to BKG. The individual was lying in a tightly flexed position on its left side on N-S axis with its head to S. The remains were not accompanied by any artifacts.

The remains belonged to 40-50 year old male.

The absolute date of the feature is 4450BC (93.5%) 4320 cal BC [10].

Petrous part of temporal bone was sampled for our study.

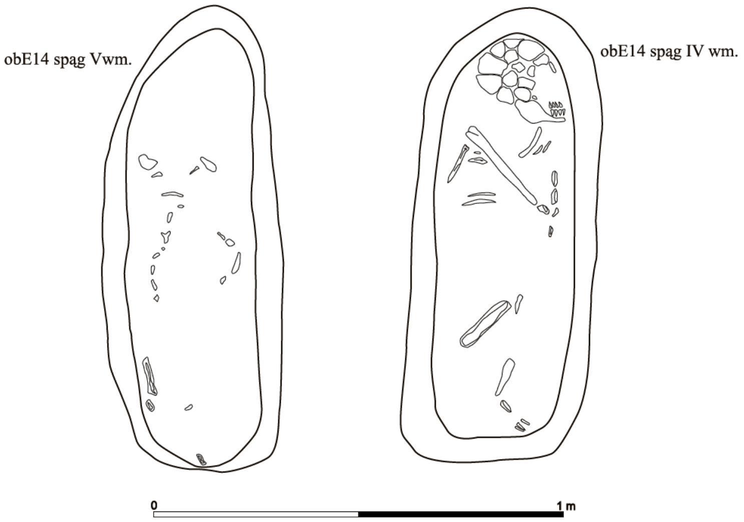

Figure 21. The burial Feature no. E14 from Ludwinowo 7 site [10].

5. LDN Malice Culture (n = 1)

5.1. Kazimierza Mała (n = 1, KM1)

Kazimierza Mała is a site with several occupation phases and was excavated in 1998-9 and 2000-02. The Malice Culture settlement where two burials were found was located on top of earlier LBK settlement. We were only able to reach and analyze one of the burials (Feature 2).

The individual was lying in N-S torso was lying on its back while legs and arms were flexed and the head in the S was facing W.

The remains were accompanied by 4 ceramic vessels. One of the vessels was a cup with stroked ornament, one was ornamented with several lines of fingernail imprints, and the other two were so called bath-shaped vessels.

The remains were interpreted as belonging to an adult woman [11].

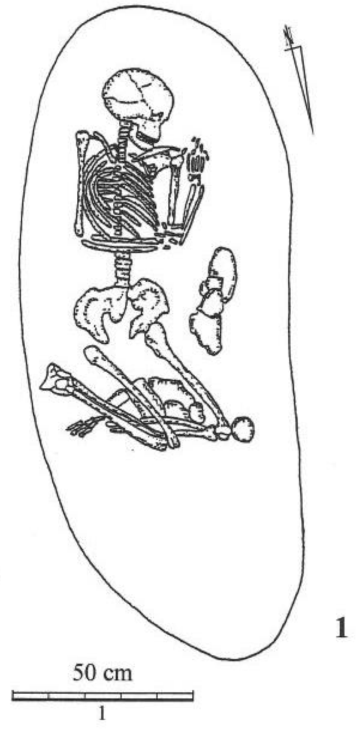

Figure 21. The burial Feature no. 1 from Kazimerza Mała [11].

References:

1. Barska K, Migal W, Ciura M. Człowiek z Janisławic. Historia pewnego archeologicznego odkrycia. In: Brzeziński W, editor. Prehistoryczny Łowca Wyst. O Człowieku Z Janisławic. Warszawa; 2013. p. 3–16.

2. Stanaszaek Ł, Mańskowska-Pliszka H. Nowe spojrzenie na człowieka z Janisławic. Analiza antropologiczna-kliniczna szkieletu. In: Brzeziński W, editor. Prehistoryczny Łowca Wyst. O Człowieku Z Janisławic. Warszawa; 2013. p. 17–26.

3. Czekaj-Zastawny A, Piotr M, Valde-Nowak, Paweł. Kultura ceramiki wstęgowej rytej. In: Czekaj-Zastawny A, editor. Obrządek Pogrzebowy Kult. Pochodzenia Naddunajskiego W Neolicie Pol. Płd.-Wschod. 56005500-2900 BC. Kraków: IAE PAN; 2009. p. 175–216.

4. Sobkowiak-Tabaka, Iwona, editor. Osadnictwo pradziejowe na stanowisku nr 13 w Kruszynie, gm. Włocławek, woj. Kujawsko-Pomorskie. in press.

5. Kaczanowska M, Tunia K. Kultura lendzielska. In: Czekaj-Zastawny A, editor. Obrządek Pogrzebowy Kult. Pochodzenia Naddunajskiego W Neolicie Pol. Płd.-Wschod. 56005500-2900 BC. Kraków: IAE PAN; 2009. p. 259–308.

6. Bednarczyk J. Z badań nad zespołem osadniczym ludności z kręgu kultur ceramiki wstęgowej w Kruszy Zamkowej, stan. 3, woj. Bydgoszcz (część sepulkralna). Archeol. Pol. 1980;32:55–83.

7. Mucha E, Piontek J, Otocki P. Analiza antropologiczna neoliycznych szkieletów z Kruszy zamkowej, stan. 3, woj. Bydgoszcz. Archeol. Pol. 1980;32:85–80.

8. Czerniak L. Brześć Kujawski type settlements at Polish Lowlands. Archeol. Rozhl. 2002;54:1–14.

9. Czerniak L, Marciniak A, Bronk Ramsey C, Dunbar E, Goslar T, Barclay A, et al. House time: Neolithic settlement development at Racot during the 5th millennium CAL B.C. in the Polish lowlands. J. Field Archaeol. 2016;41:618–40.

10. Sobkowiak-Tabaka, Iwona, editor. Osadnictwo pradziejowe inowożytne na stanowisku 7 (AUT 112) w Ludwinowie, gm. Włocławek, woj. Kujawsko-Pomorskie. in press.

11. Kadrow S, Czerniak L, Dobrzańska, Halina, Golański, Adam, Grabowska, Beata, Kurgan-Przybylska, Małgorzata, et al. Kultura malicka. In: Czekaj-Zastawny A, editor. Obrządek Pogrzebowy Kult. Pochodzenia Naddunajskiego W Neolicie Pol. Płd.-Wschod. 56005500-2900 BC. Kraków: IAE PAN; 2009. p. 217–58.
