# Supplementary figures and images for: Late Danubian mitochondrial genomes shed light into the Neolithisation of Central Europe in the 5th millennium BC
Source: BMC Evol Biol. 2017 Mar 16;17:80. doi: 10.1186/s12862-017-0924-0 (PMC5356262; doi:10.1186/s12862-017-0924-0)

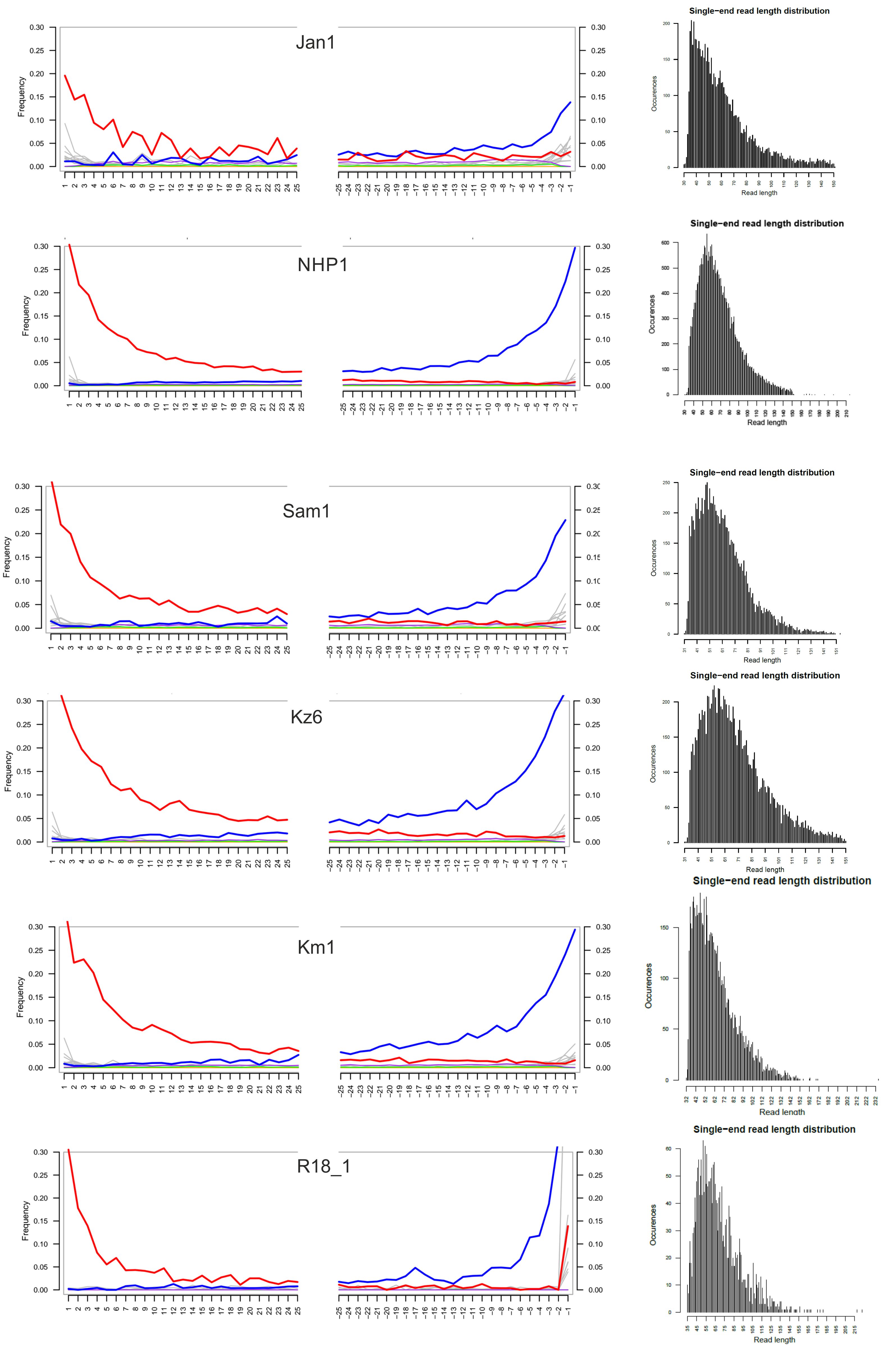

Supplement: Additional file 3: Figure S1. — Deamination patterns: frequency of C to T transitions (red) at the 5′ends of reads (left) and frequency of G to A transitions (blue) at the 3′ends of reads (middle). and fragment length distribution of sequenced libraries (right). (JPG 974 kb) [file 12862_2017_924_MOESM3_ESM.jpg]
